# Presence of tumor DNA in aqueous humor is correlated with high risk uveal melanoma

**DOI:** 10.1038/s41598-025-03915-7

**Published:** 2025-06-03

**Authors:** Nicole Barwinski, Mael Lever, Philipp Rating, Leyla Jabbarli, Miltiadis Fiorentzis, Nikolaos E. Bechrakis, Dietmar R. Lohmann, Michael Zeschnigk, Claudia H. D. Le Guin

**Affiliations:** 1 Institute of Human Genetics, University Hospital Essen, University Duisburg-Essen, Essen, Germany; 2 Department of Ophthalmology, University Hospital Essen, University Duisburg-Essen, Essen, Germany; 3 German Cancer Consortium (DKTK), partner site Essen/Düsseldorf and German Cancer Research Center (DKFZ), Heidelberg, Germany

**Keywords:** CfDNA, Liquid biopsy, Prognostic testing, Uveal melanoma, Ocular liquids, Aqueous humor, Biomarkers, Vitreous body, Cancer, Genetics

## Abstract

Metastatic risk stratification is critical for uveal melanoma (UM) management, as approximately up to half of patients develop metastatic disease. Current prognostication for patients undergoing eye-preserving therapies relies on tumor staging and molecular analysis of tumor tissue obtained through potentially invasive biopsy, which can be challenging. While liquid biopsy using cell-free DNA (cfDNA) has emerged as a less invasive alternative for other cancers, studies have shown limited utility of blood-derived cfDNA in UM due to low tumor DNA fractions. This study investigates the potential of aqueous humor (AH) and vitreous body (VB) aspirates as alternative sources of tumor DNA for molecular prognostication in UM patients at the time of diagnosis. In this prospective study, AH and/or VB samples were collected from 96 consecutive UM patients undergoing enucleation, transretinal endoresection or transretinal biopsy. DNA was extracted from the ocular fluids and analyzed for the presence of tumor-derived DNA using deep amplicon sequencing targeting mutations in *GNAQ* and *GNA11*. This approach achieved an average read depth of 120,000, enabling highly sensitive detection of tumor-specific variants. Tumor DNA was detected in at least one ocular fluid (AH or VB) in 43 of 88 evaluable patients (49%), with variant allele fractions (VAFs) ranging from 0.3 to 50%. Of these positive cases, tumor DNA was identified in VB only in 22 patients, AH only in 5 patients, and both fluids in 16 patients. Importantly, tumor DNA in AH was almost exclusively observed in patients with monosomy 3 UM. No significant correlation was found between the presence of tumor DNA in either ocular fluid and primary tumor size or location. Liquid biopsy of AH and VB offers a promising, minimally invasive strategy for obtaining tumor DNA in nearly half of UM patients at diagnosis. The strong association between detectable tumor DNA in AH and monosomy 3 status warrants further investigation and may offer valuable insights into UM biology and dissemination mechanisms. This approach may improve risk stratification and inform personalized treatment strategies for patients with UM.

## Introduction

Uveal melanoma (UM) is the most common malignant primary intraocular tumor in adulthood. Age-adjusted incidence accounts for 5.2 cases per million in the United States and ranges from two to more than eight cases per million in Europe^1^. Although the prognosis for early-stage UM is generally favorable, the disease metastasizes to distant organs in about 50% of patients, where it poses significant treatment challenges and is often associated with poor survival prospects^2^. At the time of primary tumor diagnosis, distant metastasis is present in fewer than 4% of patients^3^.

Uveal melanoma (UM) can be classified into two main molecular subtypes with distinct metastatic potentials. This classification strongly predicts the lifetime risk of developing metastatic disease. Prognostic tests for metastatic potential often determine the ploidy status of chromosome 3 as a well-established prognostic biomarker^4,5^. Other molecular features for differentiating the UM classes such as the gene expression profile (GEP) or the mutational landscape covering the UM class specific, recurrently mutated genes *EIF1AX*, *SF3B1* and *BAP1*, are also used as prognostic markers^6,7^. While current treatment strategies for UM are not directly guided by molecular prognostication, knowledge of individual risk can significantly impact patient counseling and reduce anxiety associated with prognostic uncertainty. Many patients prefer knowing their prognosis, even if unfavorable, rather than remaining uncertain about their future^8,9^. Diagnosis of intraocular tumors can often be made by clinical examination and non-invasive imaging techniques^10,11^. In rare cases with uncertain clinical diagnosis, a small biopsy may be necessary to confirm UM. Furthermore, a more precise prognostication can help identify patients to be included in adjuvant treatment studies in the near future.

Detection of UM-specific *GNAQ/GNA11* gene mutations, present in over 92.5% of UM^12,13^, is a reliable diagnostic biomarker distinguishing UM from non-melanocytic lesions.

Currently, prognostic testing and molecular classification of UM rely on tumor tissue typically obtained through enucleation, transretinal biopsy, endoresection, or transscleral resection. Patients undergoing eye-preserving treatments like brachytherapy or proton beam radiotherapy require a pre-treatment biopsy, which introduces risks such as bleeding, endophthalmitis, or retinal detachment. Consequently, there is a significant need for less invasive methods to obtain tumor DNA. Cell-free DNA (cfDNA) has emerged as a promising source of tumor biomarkers in UM, offering potential for prognostication, early metastasis detection, and treatment monitoring^14–17^.

Most studies have utilized deep amplicon sequencing or digital droplet PCR to detect circulating tumor DNA (ctDNA) in UM based on detection of *GNAQ/GNA11* gene mutations^15,18^. A previous large cohort study (*n* = 135) employing ultra-deep amplicon NGS found limited utility of blood testing at the time of primary diagnosis due to low ctDNA abundance^19^. However, at later time points a substantial increase in blood ctDNA was observed in 17 of 21 patients who subsequently developed metastases. Similarly, another study detected ctDNA in the blood of 31% of patients undergoing radiotherapy, albeit at low proportions (0.24 − 2%)^17^.

Aqueous humor has proven a valuable source of tumor-derived DNA in retinoblastoma, and studies on a few patients have demonstrated the presence of detectable ctDNA in the aqueous humor of UM patients as well, enabling downstream genetic analysis^20^. DNA concentrations varied based on clinical factors such as tumor location (ciliary body vs. choroidal) and treatment status, with lower levels observed before treatment^20,21^. While vitreous aspirate represents another potential source of liquid biopsy material in ocular oncology, its utility in UM remains unexplored.

In this study, we evaluated the potential of aqueous humor and vitreous aspirates as sources of tumor-derived DNA at the time of diagnosis in a large cohort of 96 uveal melanoma patients. As ocular fluid sampling is a less-invasive approach, it offers a significant advantage over traditional tissue biopsies to obtain tumor.

## Methods

### Patient cohort

A total of 96 consecutive patients who agreed to participate were included in this prospective study conducted between August 2022 and January 2023. Only patients who had aqueous humor and/or vitreous aspirate as a by-product of treatment were included. In 50 (57%) of these patients, the specimen was obtained from the enucleated globe, in 33 (38%) patients a specimen was obtained prior to surgical local resection of the irradiated UM. In 7 patients (8%), a vitreous specimen was obtained prior to transretinal tumor tissue biopsy. In 4 cases (5%), secondary enucleation was performed months after previous treatment, and specimens were obtained from the enucleated globe. The remaining two of the 96 patients had previously been treated at another ocular oncology department and detailed information on their treatment was not available. We received aqueous humor from 84 patients and vitreous body aspirate from 92 patients. Both specimens were available from 80 patients, while only the aqueous humor was available from 4 patients and only the vitreous body from 12 patients. The study was conducted in accordance with the Declaration of Helsinki, and ethical approvals have been granted by the local ethics committee. Written informed consent to participate in this study was given by all 96 patients.

### Sample collection

After enucleation, aqueous humor was obtained by puncturing of the anterior chamber with a 23 gauge needle. Vitreous body was then obtained from the contralateral side of the tumor via puncture with a new 23-gauge needle, followed by the aspiration of approximately 500 µl of fluid. In case of cataract surgery approximately 200 µl aqueous humor was obtained through a 1 mm paracentesis before injection of a viscoelastic substance and removal of the intraocular lens. For vitrectomies with transretinal biopsy or transretinal endoresection a vitreous sample of approximately 500 µl was collected by the vitreous cutter prior to the surgically contact with the tumor. Each sample was collected as by-product. Aqueous humor, vitreous body aspirate and tumor tissue samples were collected in 2 ml screw cap tubes, kept in liquid nitrogen for transportation and stored at -80 °C. Additionally, tumor tissue was collected in terms of routine diagnostics.

### DNA isolation

Vitreous body aspirates were pre-processed by cryogenic grinding. Samples were removed from the mortar by rinsing with 2 ml PBS. DNA was isolated from both specimens using the QIAmp Circulating Nucleic Acid Kit (Qiagen) with the protocol for 2 ml and 1 ml volume for vitreous body aspirate or aqueous humor samples, respectively, where dPBS was added to achieve the required volume. DNA from tumor tissue was isolated as described^22^.

### Sanger sequencing

Sanger Sequencing of *GNAQ* and *GNA11* codons 209 and 183 was performed on DNA isolated from primary tumor tissues. First, a PCR was performed using 12,5 µl Qiagen Hotstar Master Mix and 400 nM of each target-specific Primer in a total volume of 25 µl (Oligonucleotides as described in^23^). The cycling conditions were 15 min at 95 °C followed by 35 cycles of 30 s at 95 °C, 30 s at 60 °C and 1 min at 72 °C followed by a final elongation at 72 °C for 10 min. PCR products were purified with ExoSAPIT™ (Thermo Fisher) and then sequenced using the BigDye^®^ Terminator v1.1 Cycle Sequencing Kit (Thermo Scientific) according to manufacturer’s instructions. The reaction product was purified using a Sephadex plate prior to Sanger sequencing on a Genetic Analyzer 3130XL (Applied Biosystems). Further data analysis was performed to identify oncogenic mutations using Geneious Prime (Version 2019) software (Biomatters).

### Ultra deep amplicon sequencing

First, the genomic regions spanning codons 209 and 183 of *GNAQ* and *GNA11* were amplified by PCR using tagged target-specific primers^23^. The reaction with a total volume of 25 µl consisted of 1x Q5 High-Fidelity Master Mix (NEB) or 1x Ultra II Q5 Master Mix (NEB), 120 nM of each primer and 5 µl cfDNA as template. The PCR products were checked via agarose gel electrophoresis prior to a second PCR amplification step using index-containing primers binding to the tags introduced in the first round PCR. The reaction contained 1x Q5 High Fidelity HotStart Master Mix or 1x Q5 High Fidelity Master Mix (NEB), 80 nM of each primer and 5 µl of the PCR product of the first PCR as template resulting in a final volume of 25 µl (for Oligonucleotide sequences and PCR conditions see^23^). The PCR product of the second PCR was also analyzed by agarose gel electrophoresis. Samples were then pooled and purified using AMPure XP Beads (Beckmann Coulter) with a bead-to-sample ratio of 1.2. The purified library was eluted in TE buffer (pH 8.0) with the elution volume depending on the number of libraries pooled and stored in a 1.5 ml screw cap tube.

Library fragment size was analyzed using a bioanalyzer (Agilent) with a High Sensitivity Chip and library was quantified by quantitative PCR using the NEBNext Library Quant Kit for Illumina (NEB) according to the manufacturer’s protocol. Libraries were denatured and diluted according to protocol and sequenced on a MiniSeq instrument using the 300-cycle MiniSeq Mid Output Kit (Illumina).

### Bioinformatical data analysis

Sequencing data from cfDNA samples were analyzed using a self-developed snakemake^24^ workflow to detect the presence and variant allele fraction (VAF) of *GNAQ* and *GNA11* oncogenic mutations. It included adapter trimming with cutadapt^25^, alignment to GRCh38 genome (release 108) with bwa-mem^26^ and mutation detection via perbase. The region of interest covers the sequence encoding triplet Q209 and R183 of the *GNAQ* and *GNA11* genes. The results of the perbase analysis were further analyzed using the R programming language. Positions with a total read depth of less than 1,000 were excluded from any further analysis. The average sequencing depth across all samples was 121,000. The threshold for mutation calling was set to a variant allele fraction of 0.3 for *GNA11* and 0.5 for *GNAQ* based on an analysis per position. Analysis was performed per position, then a consensus result per gene was generated. Ultimately, the result was compared with the mutation found in the matched tumor sample. Samples with a VAF below the threshold of 0.3 and 0.5 for the *GNA11* or *GNAQ* mutation, respectively, were classified as ”negative” and thus considered not containing tumor-derived DNA.

### Microsatellite analysis for detection of chromosome 3 status

Microsatellite analysis (MSA) for the determination of chromosome 3 status of the tumor has been performed according to^27^ and evaluated using GeneScan software (Applied Biosystems).

## Results

### Study cohort

We collected either aqueous humor or vitreous body aspirate samples or both from 96 patients with uveal melanoma. Specimens were obtained from the globe immediately after enucleation (either primary or secondary), prior to surgical resection of the irradiated UM, or prior to transretinal tumor tissue biopsy. Primary tumor tissue was available from 86 of these patients and tumor DNA was analyzed for the chromosome 3 status and for oncogenic mutations in *GNAQ* or *GNA11*. An oncogenic mutation in one of these genes was identified in 81 of the 86 samples (94%). Tumor samples that were negative for *GNAQ/GNA11* mutations by Sanger sequencing were analyzed by deep amplicon sequencing. This technique detected additional mutations in three cases with an allelic fraction of 30%, 15% and 2%.

### Presence and amount of tumor-derived DNA in ocular fluids

**Fig. 1:**
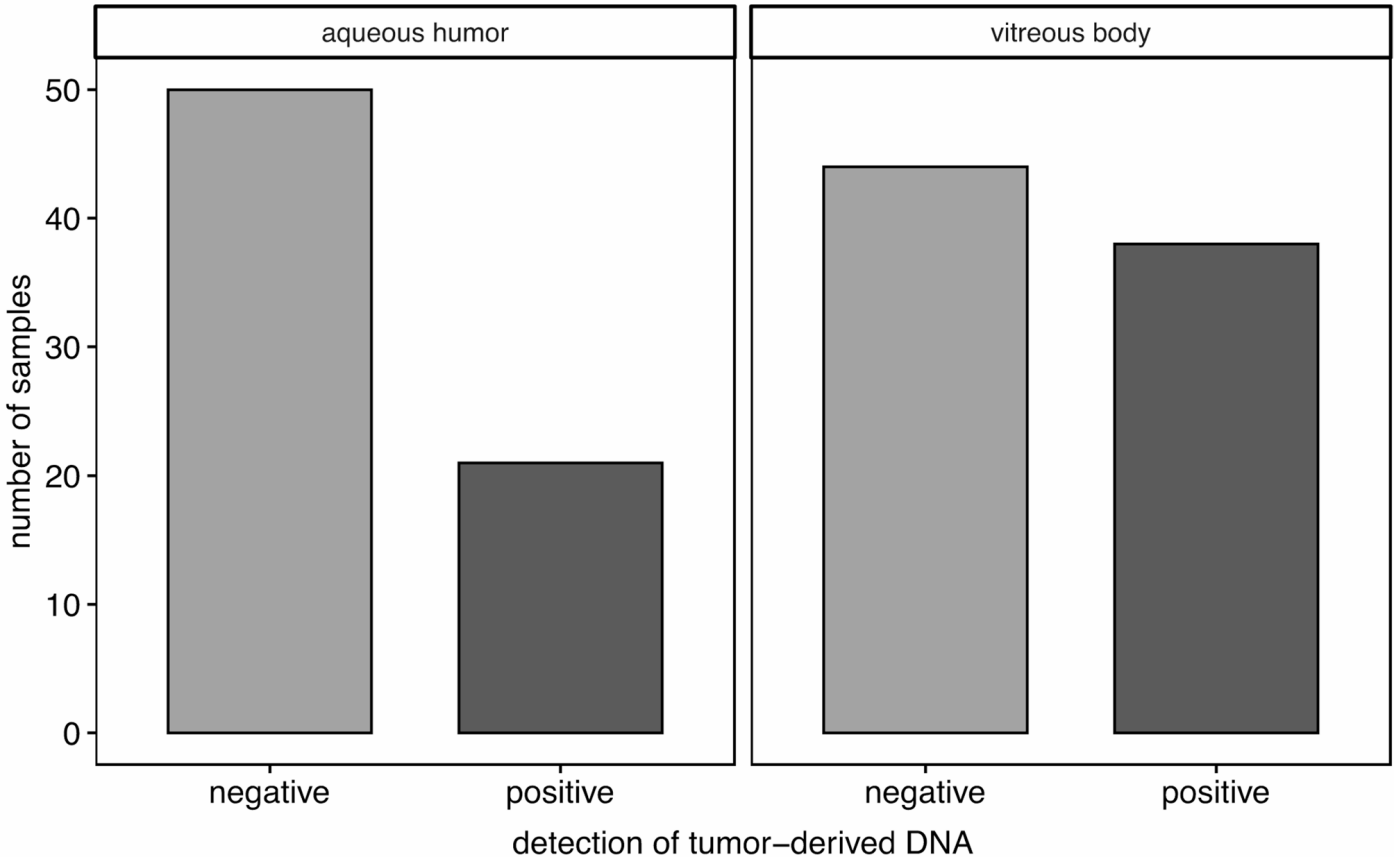
Presence (positive) or absence (negative) of tumor-derived DNA in aqueous humor or vitreous body aspirate samples as determined by targeted *GNAQ* or *GNA11* deep amplicon NGS in ocular fluids of 88 UM patients.

**Fig. 2:**
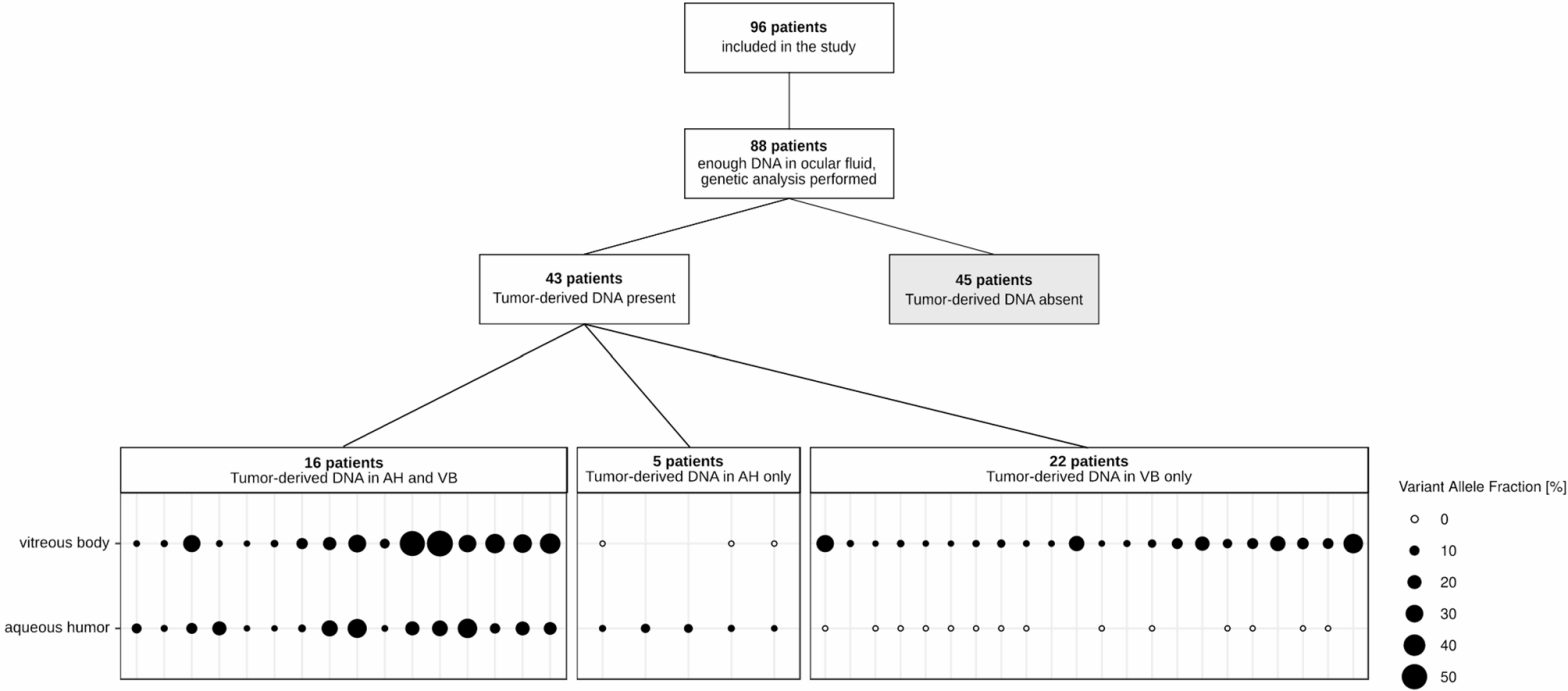
Schematic overview of patients included in this study grouped according to the presence or absence of tumor-derived DNA in the ocular fluids tested. Bubble grid chart (lower part) showing the variant allele fraction (in %) in paired liquid biopsy samples of individual patients. Absence of circle, missing data due to insufficient or missing material. Empty circle, absence of tumor derived DNA in the sample.

The *GNAQ/GNA11* mutation of the primary tumor was analyzed in all matching ocular fluids collected. If primary tumor DNA was unavailable all 4 oncogenic mutation sites were tested in ocular fluids. To achieve sensitive detection of *GNAQ/GNA11* mutations, which serve as evidence of the presence of tumor DNA, we conducted deep amplicon NGS on all ocular fluid samples from the 88 UM patients. Our results demonstrated the presence of tumor DNA in 21 aqueous humor samples and 38 vitreous body samples (Figs. 1 and 2).

In almost all cases the mutation found in the ocular fluid matched with the mutation found in the tumor. However, in three liquid biopsy cases, where the variant allele frequency (VAF) was relatively low, ranging from 0.59 to 3.6%, the oncogenic mutations identified differed from those found in the primary tumor tissue. In two of the cases, the mutation affected the same gene at the same position, yet the nucleotide substitution was distinct, resulting in an alternative amino acid substitution. In the third case, an additional nucleotide substitution was observed in the ocular fluids at position c.625, which nevertheless resulted in the same AS change at the protein level (*GNAQ*_p.Q209L). This mutation was observed in both specimens of the patient, AH and VB.

At the patient level, tumor DNA was identified in at least one of the two liquid biopsy samples in 43 out of 88 patients. In 22 of the 43 patients, tumor-derived DNA was detected exclusively in the vitreous body, in 5 cases only in the aqueous humor and in 16 cases in both samples (Fig. 2).

**Table 1:** Clinical characteristics of the study cohort.

	Tumor-derived DNA present	Tumor-derived DNA absent
	(*n* = 43)	(*n* = 45)
Age at diagnosis (years)	69.2 (50.5–92.4)	62.7 (29.3–92.9)
**Laterality**		
left eye	21	17
right eye	22	27
**Size of the tumor (mm)**		
LBD	13.8 (4.2–22.2)	14.4 (4–25)
Tumor height	9.4 (2.8–16.8)	9.3 (1.5–14.4)
**Tumor location**		
preequatorial	11	8
postequatorial	22	30
both	9	7
**AJCC status**		
stage 1	4	2
stage 2	8	5
stage 3	19	26
stage 4	9	8
**Chromosome 3 status**		
Monosomy 3	26	18
partial monosomy 3	0	2
Disomy 3	14	24
unknown	3	1
**Chromosome 8 status**		
8q amplification	15	16
normal	7	0
**Involvement of ciliary body**		
true	15	11
false	28	34

Clinical and genetic characteristics, such as tumor size and location, as well as chromosome 3 status and American Joint Committee on Cancer Classification (AJCC) score of the 88 patients analyzed, are shown in Table 1. We divided the patients into two groups depending on the presence or absence of tumor DNA in one of the two specimens, aqueous humor or vitreous body.

**Fig. 3:**
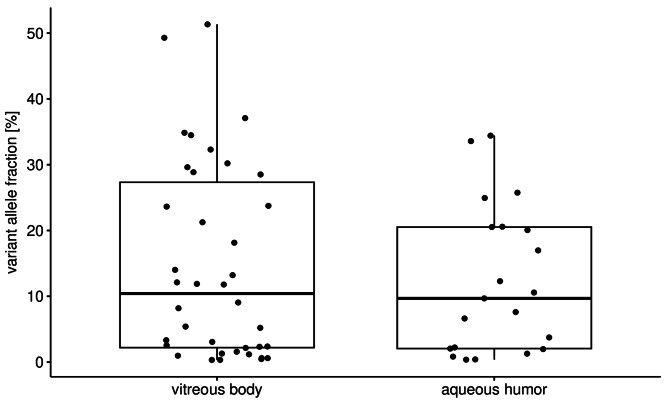
The distribution of the VAF of oncogenic *GNAQ* or *GNA11* mutations in vitreous body and aqueous humor samples that were scored positive for the presence of tumor DNA.

The tumors analyzed covered a wide range of sizes with the largest basal diameter (LBD) and tumor height ranging from 4 mm to 25 mm, and from 1.5 mm to 16.8 mm, respectively. Most of the patients had a stage 3 tumor according to AJCC classification and half of the patients (*n* = 44) had a tumor with monosomy 3. We correlated the clinical features with the tumor DNA status of the samples and found no significant association between the presence of tumor-DNA and tumor size, tumor location or patient age. However, in the ocular fluids of eyes with disomy 3, tumor DNA was more often undetected than detected (24 (63%) versus 14 (37%)), which did not reach significance (Fishers exact test p-value 0.05). The VAF of the oncogenic mutations, calculated as the proportion of mutant alleles within the wild-type alleles, can be used as a surrogate marker for the amount of tumor-derived DNA in the sample. Figure 3 shows the distribution of the variant allele fraction. The maximum VAF for mutant alleles is approximately 50% in two vitreous body samples. Assuming that the mutation is heterozygous in the tumor, this value indicates almost pure tumor DNA. The maximum VAF in the samples from aqueous humor was slightly lower with 34.4%. The median VAF for both specimens is similar with 9.7% for aqueous humor and 10.4% for vitreous body, respectively.

### Correlation of clinical and genetic features with the presence of tumor-derived DNA in ocular fluids

**Fig. 4:**
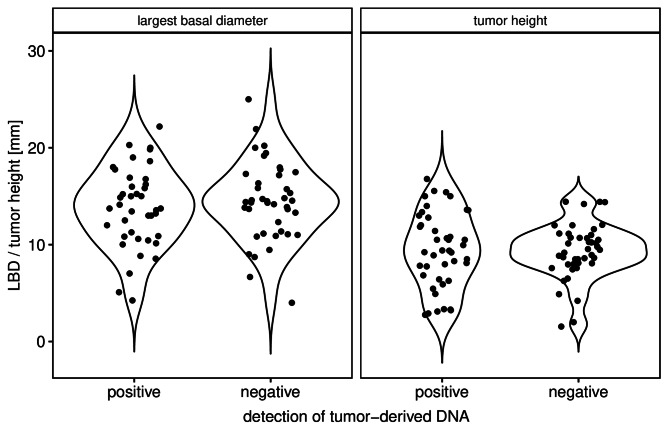
The distribution of tumor size (LBD and tumor height) is plotted for tumors showing either presence (positive) or absence (negative) of tumor-DNA in the aqueous humor or the vitreous body. (LBD, pearson correlation coefficient (PCC) = -1.34, *p* = 0.19; tumor height, PCC = 0.86, *p* = 0.39).

We tested for a possible correlation between the proportion of tumor-derived DNA and the tumor size as measured by the LBD or tumor height. Neither the largest basal diameter nor the height of the tumor showed a significant correlation with the presence of tumor-derived DNA as demonstrated in Fig. 4. The Pearson correlation coefficient between the VAF of the mutant allele and the LBD or tumor height was − 1.34 and 0.86, respectively, while the p-value was 0.19 and 0.39, respectively.

**Fig. 5:**
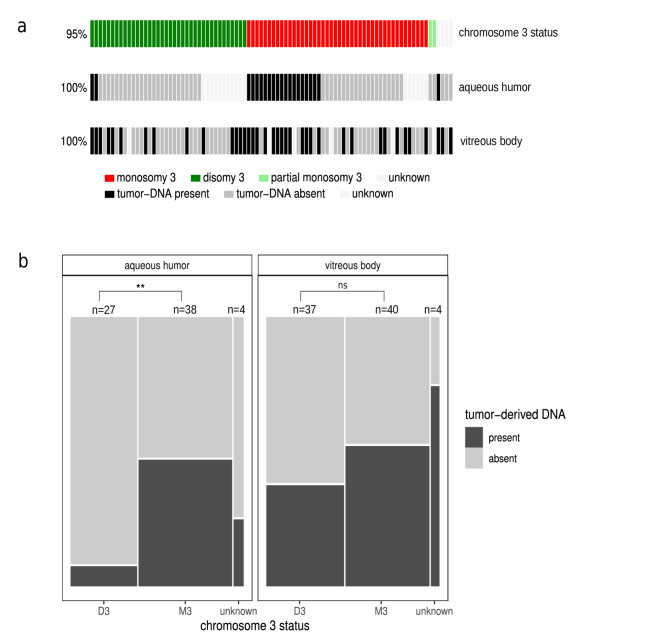
Oncoplot (**A**) and mosaic plot (**B**) showing presence of tumor-derived DNA in liquid biopsy samples stratified according to the chromosome 3 status of the primary tumor. D3: disomy 3, M3: monosomy 3, unknown: chromosome 3 status not available, ns: not significant. **: significant association between number of samples containing tumor DNA and chromosome 3 status (*p* = 0.0016).

Within our study cohort, 44 patients had a monosomy 3 tumor, while 38 patients had disomy 3 tumors. Approximately 60% of the patients with monosomy 3 tumors had tumor-derived DNA in at least one of the liquid biopsy specimens, whereas the remaining 40% did not (Fig. 5A). Overall, only 37% of patients with disomy 3 tumors had tumor DNA detected in at least one of the ocular fluids. Interestingly, when the presence of tumor DNA in the aqueous humor is correlated with the chromosome 3 status, a remarkable unequal distribution was observed, as almost exclusively DNA from monosomy 3 tumors was detectable in this ocular fluid. This difference is highly significant with a p value of 0.0016, (Fig. 5B) as calculated by fisher’s exact test. In contrast, such an uneven distribution could not be found for the tumor DNA detected in the vitreous body (*p* = 0.2564, Fig. 5).

**Fig. 6:**
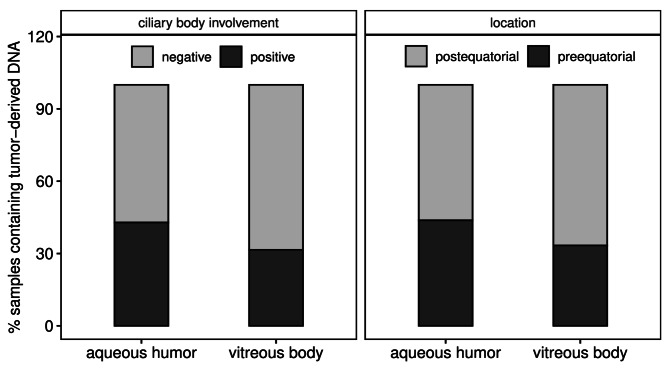
Presence of tumor-dervied DNA correlated with the tumor location. Bargraph showing the percentage of samples with presence (positive) or absence (negative) of tumor-DNA in relation to the tumor location given as either ciliary body involvement (left part) or pre/postequatorial location (right part).

Tumors with monosomy 3 are known to be more frequently associated with the ciliary body^28^ which is in a close proximity to the anterior chamber. To test whether the proximity may, at least in part, explain the preferential detection of monosomy 3 tumor DNA in the aqueous humor, we compared the location of the primary tumor (ciliary body involvement and pre-equatorial site) with the presence of tumor DNA in the aqueous humor irrespective of the chromosome 3 status. As illustrated in Fig. 6 there is no significant difference in prevalence of tumor-DNA in the aqueous humor between samples derived from tumors with or without ciliary body involvement or between those located pre- or postequatorially.

### Patients subjected to secondary enucleation

Four patients included in this study underwent secondary enucleation. The reason for the secondary enucleation was either insufficient tumor regression following the primary treatment or development of complications such as toxic tumor syndrome or radiation retinopathy (Table 2). In one of these cases, in which secondary enucleation was performed due to insufficient tumor regression, tumor-DNA was detected in the vitreous body with a VAF of 2.54%. In case 3 (Table 2) a small amount of tumor-DNA was found in the vitreous body but not in the aqueous humor. Since tumor DNA is a marker for the presence of tumor cells the proof of tumor DNA in ocular fluids months after therapy of the primary tumor may be a valuable marker for local tumor recurrence.

**Table 2:** Tumors subjected to secondary enucleations. sec. enucleation: secondary enucleation, TTT transpupillary thermotherapy.

	Primary treatment	Time interval	LBD	Reason for sec. enucleation	Tumor-derived DNA
Case 1	Brachytherapy	8 months	unknown	Insufficient tumor regression	detected (2.54%)
Case 2	Brachytherapy	6.5 years	unknown	Toxic tumor syndrome	not detected
Case 3	Brachytherapy	8 months	10.6 mm	Radiation retinopathy	detected (0.32%)
Case 4	TTT (*ex domo*)	unknown	4 mm	Insufficient tumor regression	not detected

## Discussion

At the time of diagnosis, knowledge of the metastatic risk is of utmost importance for patients with uveal melanoma. This information is crucial for both patient counseling and determining eligibility for adjuvant therapies, which are typically reserved for individuals at high risk. However, access to reliable prognostic markers is often limited in patients undergoing eye-preserving therapy as current prognostication relies on tumor tissue obtained through enucleation, surgical resection, or biopsy, each with potential complications. While cell-free tumor DNA (ctDNA) in blood offers a promising alternative, it is often undetectable at time of diagnosis^19^. Sampling of ocular fluids is less-invasive and represents a potentially valuable approach, particularly for patients receiving eye-preserving therapies. This study investigated the presence and levels of tumor-derived DNA in aqueous humor and vitreous aspirates at the time of primary uveal melanoma diagnosis, an area previously unexplored.

In accordance with the study’s objective to detect tumor DNA in ocular fluids that can subsequently be utilized for downstream genetic analysis, the DNA isolation methodology was chosen to isolate DNA independent of the degree of DNA fragmentation. Accordingly, the isolated DNA sample may contain small DNA fragments resulting from apoptotic processes (such as cfDNA) as well as unspecifically degraded genomic DNA.

We were able to show that tumor-derived DNA can be detected and isolated from ocular fluids in nearly half of the UM patients tested at time of diagnosis. Ethical considerations restricted the inclusion of patients to those whose ocular fluids could be obtained as a by-product of treatment, thus introducing a selection bias in our cohort. Consequently, smaller tumors typically managed with eye-preserving therapies are underrepresented. Despite this limitation, we found no correlation between tumor size and the presence of tumor DNA in ocular fluids, suggesting that even smaller tumors may shed detectable amounts of tumor DNA in ocular fluids.

Mutual exclusive oncogenic *GNAQ* and *GNA11* mutations are highly characteristic for UM affecting > 90% of primary tumors. The availability of matched primary tumor tissue in most of our cases facilitated targeted mutation analysis in the corresponding ocular fluids in the present study. In a clinical setting where tumor tissue is unavailable, comprehensive screening for all oncogenic mutations in both paralog genes *GNAQ* and *GNA11* as well as in *CYSLTR2* and *PLCB4*, would be necessary to improve the detection rate of tumor-derived DNA in ocular fluids. In the present proof of principle study the later genes were not examined as these somatic mutations are rarely observed. In most patients, the VAF which is given by the number of mutant reads divided by the number of wild type reads is a measure of the proportion of tumor DNA, but in few patients rare homo- or hemizygosity of the mutation, tumor heterogeneity or the absence of such a mutation may impair this correlation.

In three of 96 patients, we observed discordant mutation calls between DNA from primary tumor tissue and ocular fluids. This may reflect tumor heterogeneity, as analysis of a limited tumor sample may not fully represent the mutational landscape of the entire tumor. Co-occurrence of different *GNAQ* mutations within the same tumor, while rare, has been previously reported^29,30^.

Prior studies have investigated ctDNA in plasma and aqueous humor of UM patients before, during, and after radiotherapy^17,20^. One study detected plasma ctDNA in 31% of patients undergoing brachytherapy, with variant allele frequencies (VAFs) ranging from 0.24 to 2%^17^. Such low ctDNA abundance poses a significant challenge for downstream genetic analyses. In contrast, in AH and VB we observed substantially higher VAFs (up to 50%), with detectable tumor DNA in approximately half of our cohort. The higher abundance facilitates downstream analyses, including mutation profiling by panel sequencing and assessment of prognostically relevant copy number variations (CNVs)^20^. Therefore, for prognostication at the time of diagnosis or treatment, ocular fluid appears to be a more promising source of tumor DNA than plasma for patients undergoing eye-preserving therapies.

A remarkable, albeit preliminary result of this study was that tumor DNA in aqueous humor was predominantly derived from M3 tumors which have a very high risk for metastatic progression. While this finding requires validation in a larger cohort (and the reported p-value should be interpreted cautiously due to the lack of multiple testing correction), it suggests a potential link between tumor biology and DNA shedding into the aqueous humor. Notably, the presence of tumor DNA in aqueous humor did not correlate with tumor proximity to anterior chamber, further supporting a biological rather than a simple anatomical explanation. The mechanism underlying this preferential detection of M3 tumor DNA remains unclear. Malignant UM cells, which are characterized by high metastatic potential, are capable of cell invasion and extravasation^31,32^. These properties may allow the UM cells to leave the tumor, entering the aqueous humor and carry tumor DNA into this compartment. Interestingly, the two disomy 3 (D3) tumors that were positive for tumor DNA in aqueous humor also exhibited high-risk features, with one of them demonstrating extraocular growth and the other presenting with a large basal diameter (15 mm)^27,33,34^, thus providing further evidence that tumors associated with a poor prognosis are more likely to shed DNA into the AH. If this correlation is confirmed in more extensive studies, the presence of tumor DNA in aqueous humor could represent a novel, independent prognostic marker.

## Conclusion

The isolation of tumor DNA from ocular fluids for genetic analysis is a less invasive procedure than traditional tissue biopsies. The objective of this study was, therefore, to evaluate aqueous humor or vitreous body aspirates as a potential source of tumor-derived DNA at the time of diagnosis in a large cohort of 96 uveal melanoma patients. In approximately half of the patients, sufficient levels of tumor-derived DNA were identified at the time of diagnosis for downstream genetic analyses and prognostic testing in at least one of the ocular fluids tested. The presence of tumor DNA in the aqueous humor correlated with the chromosome 3 status of the primary tumor, suggesting that tumors associated with high metastatic risk are more likely to shed DNA into this ocular fluid.

## Data Availability

The datasets generated and/or analysed during the current study are available in the European Archive repository, [Accession number PRJEB83883].

## Abbreviations

UM: Uveal melanoma
GEP: Gene expression profile
AH: Aqueous humor
VB: Vitreous body aspirate
cfDNA: Cell-free DNA
ctDNA: Cell-free tumor DNA
VAF: Variant allele fraction
MSA: Microsatellite analysis
NGS: Next generation sequencing
LBD: Largest basal diameter
M3: Monosomy 3
D3: Disomy 3
TTT: Transpupillary thermotherapy
CNV: Copy number variations
